# Factors Influencing Consumers’ Organic Food Continuous Purchase Intentions during the Post-Pandemic Era: An Empirical Investigation in China

**DOI:** 10.3390/foods12081636

**Published:** 2023-04-13

**Authors:** Xin Qi, Junjie Mou, Chaoyue Meng, Angelika Ploeger

**Affiliations:** 1 Management College, Ocean University of China, Qingdao 266100, China; 2 Specialized Partnerships in Sustainable Food Systems and Food Sovereignty, University of Kassel, 37213 Kassel, Germany

**Keywords:** organic food, Chinese consumer, continuous purchase intention, COVID-19, M-TPB

## Abstract

During the evolutionary phases of the COVID-19 pandemic, consumers’ eating habits and spending behaviours have progressively shifted to the pursuit of the safer and healthier food products, such as organic food. Therefore, this study investigated the factors affecting Chinese buyers’ organic food continuous purchase intentions (CPI) during the post-pandemic era. To better adapt to the current consumption context in China, this study proposed a modified TPB framework (M-TPB), by replacing subjective norms with Chinese cultural variables, such as face consciousness and group conformity, and adding constructs of perceived value of organic food (PVOF), health consciousness, and the impact of COVID-19 (IOC). Convincingly, experimental results from a structural equation model analysis of 460 usable responses indicate that M-TPB has superior explanatory power (R^2^ = 65%) compared with the TPB model (R^2^ = 40%) for explaining organic food CPI during the post-pandemic period. The path analysis demonstrated that perceived behavioural control, attitude, face consciousness, group conformity, health consciousness, IOC, and PVOF had substantial positive effects on CPI, while subjective norms were not significantly related. Moreover, IOC exhibited a positive and significant relationship with health consciousness and PVOF. These findings can be useful for stakeholders in the Chinese organic food industry to generate timely promoting strategies during the post-pandemic period.

## 1. Introduction

The coronavirus disease 2019 (COVID-19) disrupted people’s habits and lives. The latest numbers of infections and deaths in the world’s population are staggering. To date, pandemic figures have reached more than 750 million cases and more than 6.8 million deaths [1]. On 8 January 2023, three years after adopting a relatively strict ‘dynamic clearing’ epidemic prevention policy, the Chinese government recently loosened the COVID-19 measures in a major policy shift. At the same time, however, increasing numbers of people have recently been infected with COVID-19, which has been considered a long-term crisis without a foreseeable end date [2,3]. The lifestyle habits of billions of people, including physical activity levels and dietary habits, have drastically changed during the COVID-19 pandemic [3,4]. The pandemic has stimulated consumers’ interests in safety and health-related attributes of food, pushing them to reconsider all possible ways that the global food supply chain can satisfy the current needs [5,6,7,8]. Consequently, organic food products, which have healthy, safety, and environmental-oriented characteristics, have been noticed and chosen more frequently chosen by consumers since the developing phases of the pandemic [5]. During the pandemic, the global market retail sale of organic food dramatically increased, from $115 billion in 2019 [9] to $136 billion in 2021 [10]; this is an increase of 18.3% in two years. It is therefore essential for those involved in the organic food sector to adjust their business objectives and capabilities to meet the current demands and significant growth occurring during the post-pandemic periods.

Organic food in China refers to agricultural and related products manufactured and processed in an organic manner, without using synthetic chemical herbicides, veterinary medicines, feed additives, or genetic technology; these products must also meet organic requirements and standards and have been certified organic by an accredited body [11]. As the world’s fourth-largest organic market, organic food consumption and production in China has expanded rapidly over recent years [12]. Due to increasing concerns about safety and health issues during the pandemic, the demand for organic food products is expected to continue, and the market will continue to grow [13]. Certain organic retailers also mentioned that online sales of organic food increased between 30 and 50 percent during the lockdown period [14]. The Chinese official statistics showed that, in 2021, there were 23,617 organic-food-labelled products, 14,847 organic food enterprises, and $15.83 billion in domestic sales, with an 18.3% increase over 2020 [15]. Although there has been remarkable development in Chinese organic food markets, they make up less than 1% of the total food market [16]. Therefore, it is important to explore the reasons hindering the development of China’s organic market and investigate the underlying factors influencing consumers’ purchases; these have the practical research significance of responding to the great potentiality occurring during the pandemic era. 

Several different theories have been put forward as explanations for the behaviour of individuals. Among them, the theory of planned behaviour (TPB), proposed by Ajzen [17], has been broadly and successfully applied in food choice studies [18,19,20]. However, the TPB has also been criticized for several reasons, such as debates regarding the efficacy of the original constructs and inadequate attention paid to other human behavioural variables; it suggested that these evolve and adjust in different contexts and behavioural scenarios. To be more adaptable to the Chinese context, Qi and Ploeger (2019) [21] modified the TPB by introducing two Chinese cultural elements (i.e., group conformity and face consciousness) to substitute for the primary variable of subjective norms. Although they have successfully applied their model to clarify consumers’ willingness to buy green food in Qingdao, China, scholars still suggest that the model’s effectiveness should be tested in different periods and conditions. Given that the similarities between the characteristics of organic food and green food, we referred to their approach of incorporating Chinese cultural characteristics and examined the model’s applicability in the context of purchasing organic food products during the post-pandemic period. 

Beyond the cultural influences, other salient aspects related to the pandemic and organic food purchasing behaviours should be considered when exploring current organic food purchase behaviours. The pandemic situation has also momentously influenced consumers’ attitudes towards safety and health matters and food behaviour. Maintaining a healthy lifestyle and consuming a safe diet can support an individual’s healthy immune system in the fight against viral infections [22,23]. Thus, considering the tremendous effect of COVID-19, our study attempts to extend TPB by incorporating the construct of the impact of COVID-19 (IOC) to explore its specific influence on Chinese consumers’ organic food purchasing behaviours. Moreover, research conducted during the COVID-19 pandemic seems to support the hypothesis which assumes that health consciousness and perceived value of organic food (PVOF) influence eating and buying habits [24,25], while the key influencing mechanisms between IOC and these two variables are in a state of ambiguity. 

Furthermore, prior research has primarily focused on investigating organic or green purchase intention during the pandemic [5,26]. However, to the best of our knowledge, the existing research does not examine consumers’ post-adoption behaviour, or more specifically, their continued purchase intentions for organic products. The current trend has shown increased consumer interest in purchasing safer and healthier food commodities during the different COVID-19 pandemic stages [8]. However, organic food continuous purchase intention with respect to a pandemic scenario has not been significantly addressed; specifically, no study on this topic has been conducted in the Chinese context. Consumers’ continued purchase intention for organic food also serves as a signal that the organic food industry has the potential to generate profit and be sustainably competitive, which can create immense economic and practical value for the survival and growth of entities in the Chinese organic industry. Therefore, this study attempts to fill the above-mentioned gaps by exploring the factors that induce Chinese consumers’ organic food continuous purchase intentions during the post-pandemic stage.

The present study aimed to establish an appropriate framework to investigate which elements affect Chinese consumers’ organic food continuous purchasing intentions during the post-pandemic era. Based on the TPB, we have developed a modified theory of planned behaviour (M-TPB) model by inserting certain important factors related to the pandemic and the research context; furthermore, we used structural equation modelling (SEM) to compare models and examine how each construct performed. This research fills the research gap regarding continuing purchase behaviour for organic food during the evolutionary phases of the pandemic in the Chinese context and provides timely and practical revolutionary inspiration for key stakeholders to further stimulate organic food consumption in China.

## 2. Theoretical Framework and Development of Hypotheses

### 2.1. Theoretical Framework

**Figure 1:**
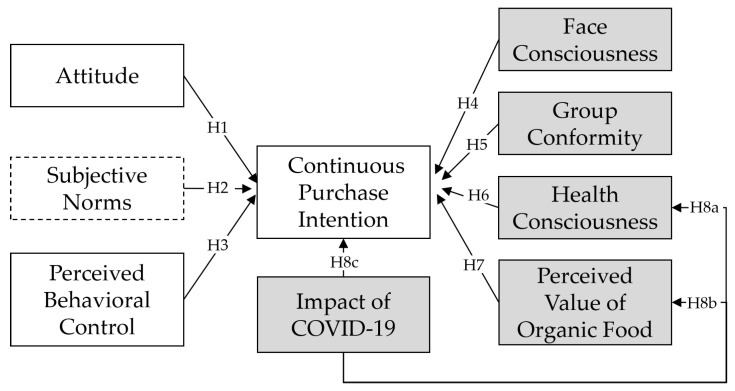
Conceptual Model. Note. Constructs derived from the original TPB model are demonstrated in white blocks; the white and grey solid blocks are the variables in the M-TPB model, and the grey blocks are the additional constructs based on the original TPB model; H1, Hypothesis 1; H2, Hypothesis 2; H3, Hypothesis 3; H4, Hypothesis 4; H5, Hypothesis 5; H6, Hypothesis 6; H7, Hypothesis 7; H8a, Hypothesis 8a; H8b, Hypothesis 8b; H8c, Hypothesis 8c.

To better understand Chinese consumers’ organic food continued purchase intentions during the post-pandemic period, we applied TPB and then proposed a modified theory of planned behaviour (M-TPB) model by inserting the cultural constructs of face consciousness and group conformity, removing the original construct of subjective norms and adding three constructs (i.e., health consciousness, PVOF, and IOC). The research structure is demonstrated in Figure 1. 

### 2.2. Development of Hypotheses

#### 2.2.1. Constructs from the TPB

- Attitude

As Ajzen [17] stated, attitude measures individuals’ positive or negative evaluation with regard to the behaviour under discussion. A recent study revealed that attitudes towards sustainable behaviours that are related to the climate and environment have the greatest influence on the intention to alter one’s behaviours around nature and environmental conservation [27]. In terms of green and organic product consumption, previous empirical studies demonstrated that consumers’ attitudes and purchase intentions are positively and significantly correlated [28,29]. For example, Wongsaichia et al. [30] recently investigated green food consumption with 500 consumers in Thailand, and their results confirmed that attitudes have significantly contributed to the prediction of the intention to purchase green food products. Thus, consistent with theory and the above research, our study formulated the following hypothesis:

**Hypothesis** **1** **(H1):** *Attitudes towards organic food products significantly influence Chinese consumers’ continuous organic food purchase intentions*.

- Subjective norms

Subjective norms refer to the anticipated social pressure to be involved in or not be involved in a certain action [17]. Some scholars have confirmed that, when making choices, the variable of subjective norms is a favourable antecedent that enormously influences consumers’ intentions [31,32]. For example, a study conducted in China found that the factor of subjective norms is a critical parameter that influences consumers’ buying intention towards environmentally friendly agricultural food [33]. However, other researchers [21,34] have argued for the effectiveness of subjective norms when justifying consumers’ food options and have suggested that further research is needed. Previous scholars observed an unfavourable correlation between subjective norms and green purchase intention [35,36]. Asih et al. [37] claimed that there is no significant relationship between subjective norms and interest in using green products. Therefore, based on the aforementioned discussion, this study proposes the following hypothesis:

**Hypothesis** **2** **(H2):** *Subjective norms enormously influence Chinese consumers’ continuous organic food purchase intentions.*

- Perceived behavioural control (PBC)

Perceived behavioural control can be explained as “how easy or difficult the respondent assesses the behaviour under consideration based on past experience as well as anticipated difficulties and obstacles” [17]. In the context of environmentally friendly food consumption, many researchers have identified PBC to be an essential determinant of organic food purchasing intention [21,38,39]. For instance, Boobalan et al. [40] were able to show that PBC has a favourable and powerful influence on organic food purchasing intention in both American and Indian consumers. Furthermore, a recent study conducted by Le and Nguyen [41] confirmed that PBC served as a compelling factor of organic food purchasing intention in Vietnam. Together with the previous studies, this study proposes the following hypothesis:

**Hypothesis** **3** **(H3):** *PBC has a significant relationship with Chinese consumers’ continuous intentions to buy organic food.*

#### 2.2.2. Constructs from the M-TPB

- Face consciousness

The term “face” is defined as individual’s social status or reputation when performing the particular social roles which are recognized by other people [42]. Specifically, face consciousness refers to caring about gaining, keeping, and losing face in daily lives [43]. Face culture is widespread in the Chinese culture; it significantly influences Chinese consumers’ purchase intentions and daily behaviour [44,45]. For instance, previous studies have confirmed the important impact of face consciousness on Chinese consumers’ purchase intentions towards high-quality or luxury products [46,47] and green or traceable food [21,48]. Since organic food is generally perceived as a high-quality and high-price product, conducting the organic food buying behaviour can imply a “status symbol” to make consumers gain “face”. Based on this discussion, this study proposes the following hypothesis:

**Hypothesis** **4** **(H4):** *Face consciousness has tremendous relationship with Chinese consumers’ continuous intentions towards buying organic food.*

- Group conformity

Group conformity is described as people’s behaviours which tend to be influenced by the reference group; people are inclined to imitate group members [49,50]. Past literature has confirmed a favourable correlation between group conformity and environmentally friendly purchase intentions and behaviours [21,49,51]. Compared to Western counterparts, Chinese purchasers are easily convinced by their acquaintances and tend to behave in congruent ways when making purchases. [52,53]. Notably, Thøgersen et al. [54] demonstrated that group conformity is more salient in prompting organic foods purchasing behaviours among consumers from China compared with consumers from Europe. In the domain of food consumption, Qi and Ploeger [21] discovered that Chinese people’s group conformity plays a significant and critical role in promoting consumers’ intentional process of purchasing green food. Based on the above discussion, this study presents the following hypothesis:

**Hypothesis** **5** **(H5):** *Group conformity has a tremendous relationship with Chinese consumers’ continuous intentions towards buying organic food.*

#### 2.2.3. Incorporating Additional Constructs in the TPB

- Health consciousness

As Jayanti and Burns [55] stated, health consciousness is the extent that health issues are integrated into an individual’s daily life. Many people opt to purchase organic food products as they believe that these products are healthier and contain fewer pesticide residues and synthetic chemicals than conventional products [56,57]. Numerous studies discovered conclusive evidence which suggests that health consciousness has a significant impact on consumers’ intentions to purchase organic food [24,58,59,60]. For example, the study from Parashar et al. [24] has highlighted that health consciousness was the most decisive parameter in justifying Indian customers’ buying intentions and behaviours. Accordingly, this study proposes the following hypothesis:

**Hypothesis** **6** **(H6):** *Health consciousness has a significant impact on Chinese consumers’ continuous intentions towards buying organic food.*

- Perceived value of organic food (PVOF)

Perceived value can be described as a consumer’s general assessment of the utility of a product or service, as perceived by the consumer in terms of the benefits received or the costs incurred. [61]. In the context of organic food consumption, several studies have confirmed that perceived value is an internal driver which positively influences consumers’ attitudes, intentions, and actual purchase behaviours [62,63,64,65]. According to the findings from De Toni et al. [65], perceived value has a considerable effect on repurchase intention in Brazil. In addition, the study from Lin et al. [66] revealed that two dimensions of perceived value, namely, utilitarian value and hedonic value, have significantly enhanced Chinese consumers’ continuous purchase intentions towards organic food. Based on this discussion, this study proposes the following hypothesis:

**Hypothesis** **7** **(H7):** *PVOF has a significant impact on Chinese consumers’ continuous intentions towards buying organic food.*

- Impact of COVID-19 (IOC)

The unprecedented outbreak of COVID-19 has drastically impacted people’s daily lifestyles and eating habits, especially the patterns by which people purchase and consume their food [67]. Previous studies have found that cognitive emotions caused by COVID-19 positively direct people to be more health-conscious and seek healthy foods [68,69,70]. For instance, Hu et al. [69] found that consumers’ fear of COVID-19 has a positive influence on their health concerns. This study also highlighted the strong positive relationship between the level of intolerance of uncertainty of COVID-19 and purchase intention toward green products. Similarly, Nguyen and Phan [70] showed that consumers in Vietnam with a high level of COVID-19 anxiety presented high degrees of health consciousness and purchasing intention of functional foods. Brata et al. [71] revealed that the pandemic has accelerated individual’s interest in organic food products’ attributes and values, as organic foods are generally regarded to be safer, healthier, and more pro-environmental than conventional foods [36]. Thus, we intend to investigate the impact of IOC on Chinese consumers’ PVOF, health consciousness, and continuous organic food purchase intentions during the pandemic. Accordingly, we propose the following hypothesizes:

**Hypothesis** **8a** **(H8a):** *IOC significantly influences Chinese consumers’ health consciousness.*

**Hypothesis** **8b** **(H8b):** *IOC significantly influences Chinese consumers’ PVOF.*

**Hypothesis** **8c** **(H8c):** *IOC significantly influences Chinese consumers’ continuous purchase intentions towards organic food.*

## 3. Methodology

### 3.1. Data Collection

For this study, data were gathered via a professional online questionnaire survey platform (www.wenjuan.com; accessed on 1 November 2022) to analyse the developed conceptual framework. The target population in this study was individuals living in China, a country with the world’s most prevalent consumer group. After a pretest conducted with 20 random consumers, the initial questionnaire was refined and developed to ensure its understandability and fluency. The questionnaire was distributed through WeChat, the most popular, broad-reaching social platform in China, which enables respondents to complete surveys on their mobile phones. Participation is limited to one per IP address to avoid duplicate sampling. This survey is aimed at consumers aged over 20 years, since they form the major green consumer groups in China, both existing and potential [72]. Therefore, a filter question was used to distinguish individuals whose age is less than 20 years old at the beginning of survey. The survey was available to be answered from 1 to 15 November 2022. A total of 514 questionnaires were received; 54 respondents were excluded due to the linear response patterns and item non-response (e.g., failing to complete most of the survey or not providing any demographic information), resulting in a final sample of 460 (rate of use = 89.5%). All participants gave their informed consent for inclusion, and the study was approved by our institution. Kline [73] claimed that an appropriate sample size for an investigation would be 10 cases per item. In this study, there were 27 measurement items; this gave us a minimum of 270 responses. Our sample size of 460 usable responses was therefore considered sufficient and acceptable for further data analysis. 

### 3.2. Measures

The measurements were adopted from existing papers and modified for the present study. To confirm the correct content and meaning, all items were translated into Chinese by two native speakers. The questionnaire was mainly composed of three parts. The first section introduced the purpose of our survey, as well as the definition and logo of organic food. The second section consisted of questions on behavioural attitudes, subjective norms, PBC, face consciousness, group conformity, PVOF, health consciousness, IOC, and continuous purchase intentions to organic food. A 7-point Likert scale ranging from 1 (‘strongly disagree’) to 7 (‘strongly agree’) was used to measure each of these constructs. Table 1 displays the questionnaire items and the source from which they were adopted. The third section contained five items asking for participants’ demographic information.

### 3.3. Data Analysis

This study analyses data by applying Analysis of Moment Structure (AMOS) Version 29 and Statistical Package for Social Science (SPSS) version 29. First, we applied SPSS to analyse participants’ characteristics. Secondly, AMOS was used to examine structural equation modelling (SEM) analysis in two steps. In the first step, the reliability and validity of the measurement model were tested using confirmatory factor analysis (CFA). In the second step, the full structural model was measured using *p*-values, t-values, and standardized regression coefficients (β) to assess the hypothesized relationships and the fit of the model.

## 4. Results

### 4.1. Profile of the Respondents

Table 2 illustrates general information about the sample’s demographic features. Our final sample contained more women (51.7%) than men (48.3%). Although the gender composition in our survey was slightly different from Chinese average statistics (i.e., 48.8% female; 51.2% male) [82], it is consistent with the fact that females are more willing to pay premiums for organic food than males [83]. The 20–30 age group made up most of the sample. Most respondents were married with one or more children (38.7%), 32.6% of respondents were single and 22.8% were married without children. With respect to educational level, most of our participants were highly educated, 35.9% of them had a high school or technical secondary school degree, and 46.7% of respondents had a university degree or above. This figure is higher than the average education level in China (i.e., 15.1% have high school or technical secondary school degree; 15.5% have a university degree or above) [84]. Of all participants, 47.4% of them reported that their monthly income was between 655 USD and 1310 USD.

### 4.2. Measurement Model: Reliability and Validity

The reliability and validity analysis of each measurement is displayed in Table 3. All Cronbach’s α values were greater than the acceptable limit of 0.7 [85], which means that the data of the questionnaire are sufficiently reliable. In terms of convergent validity, the composite reliability (CR) for all constructs, with values between 0.819 and 0.938, was higher than the acceptable limit of 0.6 [86]. Factor loadings were also examined for all constructs; they ranged from 0.756 to 0.944, exceeding the threshold of 0.6 [87]. In addition, the scores of the AVE (0.601 to 0.835) were higher than the recommended criterion of 0.5 [85]. Therefore, adequate discriminant validity was ensured. Considering discriminant validity, Table 4 shows that the value of the square root of the AVE was above the correlation value for each of the items. Accordingly, discriminant validity was established. In summary, the conceptual framework outlines sufficient validity (convergent and discriminant) and reliability.

### 4.3. Structural Model: Goodness of Fit Statistics

Table 5 presents structural model goodness of fit indices. As for the standard TPB model, the structural TPB model showed a good fit to the sample data, with RMSEA = 0.015, CFI = 0.998, TLI = 0.998, IFI = 0.997, GFI = 0.981 and χ^2^/df = 1.109. For the proposed modified framework (i.e., M-TPB model), its goodness of fit indices (RMSEA = 0.045; CFI = 0.973; TLI = 0.973; IFI = 0.968; GFI = 0.926; χ^2^/df =1.916) also demonstrated sufficient fit. After a satisfactory model evaluation, the M-TPB model was compared to the original TPB model. Our results show that the M-TPB model for measuring the continuous purchase intention of Chinese consumers of organic food in the post-pandemic period has a better explanatory power (R^2^ = 0.65) than the original TPB (R^2^ = 0.40). Specifically, 65% of the total variance in this study can be explained by the M-TPB model.

### 4.4. Hypothesis Testing

The proposed substantial impacts of the hypothesized paths of the TPB and M-TPB are tested and demonstrated in Table 6. With regard to the variables obtained from the TPB model, the outcomes show that Chinese purchasers’ attitudes about buying organic food (β = 0.375, t = 10.815, *p* < 0.001; β = 0.223, t = 7.647, *p* < 0.001, respectively) and PBC (β = 0.127, t =5.236, *p* < 0.001; β = 0.051, t = 2.582, *p* < 0.05, respectively) significantly and positively affect their organic food continuous purchase intention in the TPB model, as in the M-TPB, which supports H1 and H3. H2 was intended to test the significant and positive effect on consumers’ continuous purchase intentions of subjective norms. Contrary to our expectations, the path linking subjective norms to continuous purchase intentions towards organic food was not found to be significant (β = 0.057, t = 1.515, *p* > 0.05), and consequently, H2 is not supported. Regarding constructs deriving from the M-TPB, both consumers’ face consciousness and group conformity significantly influence consumers’ continuous purchase intentions of organic food, which supports H4 (β = 0.082, t = 3.315, *p* < 0.001) and H5 (β = 0.102, t = 4.768, *p* < 0.001). As proposed in H6, consumers’ health consciousness holds a tremendous and positive influence on their continuous intentions of buying organic food (β = 0.338, t = 7.861, *p* < 0.001), thus, H6 is supported. Notably, PVOF has the largest influence power (β = 0.2100, t = 5.816, *p* < 0.01) among the constructs in M-TPB. Therefore, H7 is supported. The role of IOC was found to be significantly and positively related to consumers’ health consciousness (β = 0.190, t = 5.122, *p* < 0.001), PVOF (β = 0.272, t = 6.900, *p* < 0.001), continuous purchase intentions (β = 0.091, t = 3.714, *p* < 0.001). Therefore, H8a, H8b and H8c are supported, accordingly.

## 5. Discussion

The current research investigated the elements influencing Chinese consumers’ continuous purchase intention for organic food products from the social psychological perspectives during the post-COVID-19 pandemic era. The new M-TPB model was proposed by amending and extending TPB, substituting the subjective norms into Chinese cultural elements (i.e., face consciousness and group conformity) and incorporating three new constructs (i.e., health consciousness, PVOF and IOC) into the framework.

Regarding the effects of each construct and originating from the primary TPB model on organic food continuous purchase intentions, attitude and PBC were found to positively and significantly influence consumers’ continuous intention to purchase organic food, which supports statements from previous studies [27,30,31]. It is worth mentioning that attitude has the most significant impact in both TPB and M-TPB framework, which is inconsistent with prior studies that were conducted during the different pandemic periods; those studies reported that PBC was more effective compared to other constructs [37,88]. One possible explanation for this difference is that the accessibility and convenience were perceived to be more important than buying certified food products by consumers during the early pandemic period. Nevertheless, with the adjustment of quarantine policies and the optimization of supply chain, the influencing abilities of different factors on individuals’ organic continuous buying intentions also have been changed, especially during the post-pandemic period. Therefore, promoting consumers’ attitudes towards organic food plays a critical role in boosting their continued intentions. Thus, retailers in the organic food industry should advertise the benefits of organic food to increase consumers’ perceptions and evoke positive attitudes about organic purchases. Additionally, the results of our study suggest that subjective norms failed to have a significant influence on continuous purchase intention for organic food. These findings are in line with other previous investigations [36,37] and also reinforced the arguments of the debated or poor role of subjective norms in different contexts [34].

Furthermore, the results of our investigation prove that the parameters of group conformity and face consciousness are crucial to organic food purchase intentions in the post-COVID-19 era. Accordingly, the Chinese cultural characteristics are indispensable when considering factors that drive organic food purchase intentions. These findings are consistent with the results of previous consumer behaviour studies conducted in China [89,90]. Chinese culture is based on collectivism, and Chinese people possess a greater conformity and face awareness; hence, relevant investigation or marketing policies initiated in China need to consider the cultural effects on purchase intention. Additionally, our findings highlighted that health consciousness and PVOF have a significant, strong relationship with CPI towards organic food, which supports the similar findings from prior studies [24,54]. Notably, PVOF was found to be the most momentous determinant of Chinese consumers’ organic food continued purchasing intentions in the M-TPB model. The results imply that merchants should give priority to prominently displaying the value and quality of organic food in their marketing strategies, such as emphasizing the nutritional, safe, and environmentally friendly value to increase consumers’ interest in organic foods. Meanwhile, health consciousness was also confirmed to be a critical parameter of organic food continued intentional purchasing in the pandemic period, which correlates with previous studies by Katt and Meixner [60] and Parashar et al. [24]. This shows that Chinese consumers pay close attention to their health, and their health awareness plays a significant role in their organic food purchases during the COVID-19 pandemic. As a result, marketers should effectively communicate the health-related benefits of organic products to consumers to keep their continuous purchase intentions. 

Regarding the IOC, our results revealed that IOC positively and significantly influences Chinese consumers’ health consciousness, PVOF, and continuous purchase intention. The findings showed that IOC is necessary when predicting consumers’ intentional behaviour during the post-pandemic era. Our findings indicated that IOC has a positive and significant impact on consumers’ health consciousness and PVOF. Thus, the COVID-19 period is an excellent time for key stakeholders to engage in revolutionary strategies that can propagate organic consumption in China, encouraging consumers to pay more attention to product attributes and food safety and to prefer to choose organic food. 

Lastly, regarding the overall performance of the two conceptual frameworks (i.e., TPB; M-TPB), our results showed that both models had good model fit and exhibited satisfactory explanatory power. Compared to the original TPB model, the M-TPB model was superior in explaining and predicting Chinese consumers’ organic food continued purchase intentions (the R^2^ for behavioural intention in the M-TPB model was approximately 15% higher than that in the original TPB model). Specifically, the incorporation of the Chinese cultural constructs, health consciousness, PVOF, and IOC into the original TPB model resulted in a rise in the amount of variance explaining Chinese consumers’ continued intention to buy organic food in the post-pandemic period. 

## 6. Conclusions

This study has shown the M-TPB model to be useful and comprehensive in exploring Chinese consumers’ continuous intentional processes of buying organic food products in the context of post-pandemic era. Our present research reinforces existing evidence which states that variables, including attitude, PBC, health consciousness, face consciousness, group conformity, PVOF, and IOC, have been instrumental in the intentional processes of purchasing organic food in the post-pandemic era. This study also confirmed that IOC has a significant and positive effect on PVOF and health consciousness. Although many countries around the world have loosened their pandemic control policies, and people have adapted to the existence of COVID-19, the IOC on people’s daily lives still seems to be long-lasting and continuous [2,3]. Furthermore, this study is amongst the pioneers in understanding the mechanism of forming consumers’ continuous purchase intentions from the quantitative lens. Therefore, the findings have contributed to the field of organic food purchasing behavioural research during the pandemic era and mapped a pathway for stakeholders in the organic industry to develop strategies appropriate to expanding the organic food industry in the future. Finally, given the cultural commonalities between China and other Asian countries, further studies could experiment with the application of the modified TPB model in other Asian countries.

## 7. Limitations

The current study has limitations which should be considered in future research. First, our study is limited by examining an intention stage; there is no measure of actual purchase behaviour for organic food. As there is a discrepancy between intention and behaviour [31], further research can develop our model to include purchase behaviour to strengthen present investigation results. Second, the sample bias cannot be eliminated in our survey as consumers who were not using the internet were removed from the samples. In addition, the participants in our survey had a relatively high level of education, which may have been over-represented in our research. Therefore, future research should increase the sample size and study more diverse populations. Finally, consumers’ organic food purchase behaviours, as well as consumption patterns, are expected to alter with the pandemic evolutionary phases, thus, further research could consider additional unforeseen factors throughout other pandemic phases to better explore the IOC on organic food consumption.

## Figures and Tables

**Table 1:** Questionnaire items and their source of adoption.

Variables	Items	Measurement Items	Adopted From
Continuous Purchase Intention (CPI)	CPI1	I will consider purchasing organic foods in the nearfuture.	[74]
	CPI2	I will regularly purchase organic food.	
	CPI3	I tend to buy organic food products for long term benefit.	
Attitude (AT)	AT1	I think purchasing organic food is a good idea.	[75]
	AT2	I think purchasing organic food is pleasant.	
	AT3	I think purchasing organic food is important.	
Subjective Norms (SN)	SN1	The trend of buying organic food among people around me is increasing.	[76]
	SN2	People around me generally believe that it is better for health to eat organic food.	
	SN3	My close friends and family members would appreciate if I buy organic food.	
Perceived Behavioural Control (PBC)	PBC1	It is easy for me to buy organic foods when I’m interested.	[77]
	PBC2	I possess resources, time, or opportunities to buy organic food.	
	PBC3	It is entirely up to me whether I want to buy organic food or not.	
Face Consciousness (FC)	FC1	People around me believe that buying organic food suits my identity and taste.	[43]
	FC2	Purchasing organic food can help me gain face.	
	FC3	Organic food purchase is a good way to distinguish me from others.	
Group Conformity (GC)	GC1	I may consider purchasing organic food, if people around me perceive organic food is good.	[78]
	GC2	I will purchase organic food, if people around me buy organic food as well.	
	GC3	I will purchase organic food, if I feel people around me expect me to comply with their decision to buy organic food.	
Perceived Value of Organic Food (PVOF)	PVOF1	I believe continuous consumption of organic food would promote my long-term health benefits.	[79]
	PVOF2	I believe that organic foods have higher nutritional value.	
	PVOF3	I believe organic foods have good contributions towards environmentally friendly, ecology and protects animal welfare.	
Health Consciousness (HC)	HC1	I cared about the type and amount of nutrition in the food that I consume daily.	[80]
	HC2	Organic food is benefit for individual’s health.	
	HC3	I think it is important to know well how to eat healthy.	
Impact of COVID-19 (IOC)	IOC1	I consider the coronavirus pandemic has affected me personally.	[81]
	IOC2	I consider the coronavirus pandemic has changed my consumption pattern.	
	IOC3	I consider the coronavirus pandemic has changed society.	

**Table 2:** Demographic characteristics of samples (n = 460) in comparison to Chinese average statistics.

Demographic	Variables	Frequency	Percent (%)
Gender	Male	222	48.3
	Female	238	51.7
Age	20–30	164	35.7
	31–40	109	23.7
	41–50	98	21.3
	51–60	53	11.5
	>60	36	7.8
Marital Status	Married with child or children	178	38.7
	Married	105	22.8
	Single	150	32.6
	Other	27	5.9
Education	Junior school or below	80	17.4
	High school or technical secondary school	165	35.9
	University or above	215	46.7
Monthly Income (USD)	<655	115	25.0
	655–6310	218	47.4
	>1310	127	27.6

**Table 3:** Measurement model: Reliability and validity.

Constructs	Factor Loadings		C.R		SMC		AVE		Cronbach’s a	√AVE
	TPB	M-TPB	TPB	M-TPB	TPB	M-TPB	TPB	M-TPB		
CPI			0.899	0.887			0.747	0.724	0.897	0.851
CPI1	0.833	0.840			0.694	0.705				
CPI2	0.872	0.855			0.760	0.731				
CPI3	0.887	0.857			0.787	0.735				
AT			0.888	0.888			0.727	0.727	0.886	0.853
AT1	0.794	0.792			0.631	0.627				
AT2	0.899	0.901			0.809	0.811				
AT3	0.861	0.861			0.741	0.742				
PBC			0.936	0.936			0.829	0.829	0.935	0.910
PBC1	0.875	0.875			0.765	0.766				
PBC2	0.919	0.919			0.846	0.845				
PBC3	0.936	0.936			0.875	0.875				
SN			0.819				0.601		0.740	0.775
SN1	0.772									
SN2	0.756									
SN3	0.798									
FC				0.888				0.727	0.890	0.853
FC1		0.873				0.762				
FC2		0.834				0.696				
FC3		0.851				0.724				
GC				0.938				0.835	0.938	0.914
GC1		0.944				0.890				
GC2		0.923				0.852				
GC3		0.873				0.762				
PVOF				0.867				0.685	0.867	0.811
PVOF1		0.815				0.665				
PVOF2		0.827				0.684				
PVOF3		0.841				0.707				
HC				0.850				0.653	0.848	0.808
HC1		0.792				0.627				
HC2		0.831				0.691				
HC3		0.801				0.641				
IOC				0.912				0.777	0.912	0.881
IOC1		0.913				0.834				
IOC2		0.907				0.823				
IOC3		0.821				0.674				

Note. TPB: Theory of Planned Behaviour, M-TPB: Modified Theory of Planned Behaviour; SN: Subjective Norms, AT: Attitude, PBC: Perceived behavioural Control; GC: Group Conformity, FC: Face Consciousness; HC: Health Consciousness; PVOF: Perceived Value of Organic Food; IOC: Impact of COVID-19, CPI: Continuous Purchase Intention; AVE = Average Variance Extracted (AVE = ∑SMC/(∑SMC + ∑standard measurement error), SMC = Squared Multiple Correlation (i.e., squared value of correlation between the constructs), C.R = Composite Reliability.

**Table 4:** Correlation matrix for discriminant validity.

	SN	HC	IOC	PVOFAP	PBC	GC	FC	AT	CPI
SN	0.775								
HC	0.02	0.808							
IOC	0.147	0.253	0.881						
PVOF	0.247	0.261	0.345	0.811					
PBC	0.052	0.24	0.135	0.285	0.910				
GC	0.007	0.285	0.164	0.12	0.12	0.914			
FC	0.113	0.179	0.198	0.24	0.163	0.154	0.853		
AT	0.13	0.38	0.366	0.232	0.198	0.173	0.207	0.853	
CPI	0.156	0.548	0.495	0.575	0.342	0.368	0.367	0.583	0.851

Note. The diagonal elements represent the square root of AVE; Off-diagonal elements show the correlations between constructs; SN: Subjective Norms; AT: Attitude; PBC: Perceived behavioural Control; GC: Group Conformity; FC: Face Consciousness; HC: Health Consciousness; PVOF: Perceived Value of Organic Food; CPI: Continuous Purchase Intention; IOC: Impact of COVID-19.

**Table 5:** Goodness of fit indices and explanatory power of two models.

	TPB	M-TPB	Norms
RMSEA	0.015	0.045	≤0.08 *
CFI	0.998	0.973	≥0.9 *
TLI	0.998	0.973	≥0.9 *
IFI	0.997	0.968	≥0.9 *
GFI	0.981	0.926	≥0.9 *
χ²/df	1.109	1.916	>1 and <5 *
R^2^	0.40	0.65	

Note. * Source: Bagozzi and Yi [87]; CFI = Comparative Fit Index, NFI = Normative Fit Index, TLI = Tucker–Lewis Index, GFI = Goodness of Fit Index, RMSEA = Root Mean Square Error Approximation, IFI = Incremental Fit Index.

**Table 6:** Hypotheses test results.

Hypothesized Path	Standardized Path Coefficients		t-Value		Result
	TPB	M-TPB	TPB	M-TPB	
H1: AT→CPI	0.375	0.223	10.815 ***	7.647 ***	Support
H2: SN→CPI	0.057		1.515		Not support
H3: PBC→CPI	0.127	0.051	5.236 ***	2.582 *	Support
H4: FC→CPI		0.082		3.315 ***	Support
H5: GC→CPI		0.102		4.768 ***	Support
H6: HC→CPI		0.210		5.816 ***	Support
H7: PVOF→CPI		0.266		7.883 ***	Support
H8a: IOC→HC		0.190		5.122 ***	Support
H8b: IOC→PVOF		0.272		6.900 ***	Support
H8c: IOC→CPI		0.091		3.714 ***	Support

Note. * *p* < 0.05; *** *p* < 0.001. TPB: Theory of Planned Behaviour, M-TPB: Modified Theory of Planned Behaviour; CPI: Continuous Purchase Intention, AT: Attitude, PBC: Perceived behavioural Control, GC: Group Conformity, SN: Subjective Norms, FC: Face Consciousness, HC: Health Consciousness, PVOF: Perceived Value of Organic Food, IOC: Impact of COVID-19.

## Data Availability

The data used in this study can be provided upon request.
